# Resilient Modulus Behavior and Prediction Models of Unbound Permeable Aggregate Base Materials Derived from Tunneling Rock Wastes

**DOI:** 10.3390/ma15176005

**Published:** 2022-08-31

**Authors:** Meng Wang, Qunding Yu, Yuanjie Xiao, Wenqi Li

**Affiliations:** 1 School of Civil Engineering, Central South University, Changsha 410075, China; 2 Urban Rail and Underground Engineering Design and Research Institute, China Railway Siyuan Survey and Design Group Co., Ltd., Wuhan 430063, China; 3 MOE Key Laboratory of Engineering Structures of Heavy Haul Railway, Central South University, Changsha 410075, China

**Keywords:** tunneling rock waste, unbound permeable aggregate base, gradation, repeated load triaxial test, resilient modulus, prediction model

## Abstract

Tunneling rock wastes (TRWs), which are often open- or gap-graded in nature, have been increasingly recycled and reused for sustainable construction of unbound permeable aggregate base (UPAB) courses with high porosity and desired drainability. However, there is still a lack of sufficient understanding of long-term mechanical stability of such TRW materials subjected to repeated applications of moving wheel loads. This paper aimed to characterize and predict resilient modulus (M_r_) behavior of the TRW materials used in unbound permeable aggregate base applications. To achieve this goal, five different UPAB gradations were designed based on the gravel-to-sand ratio (*G/S*) concept. In order to study their M_r_ behavior, the laboratory repeated load triaxial tests were conducted under different combinations of confining pressure and deviator stress as controlled by the levels of the shear stress ratio (SSR). The prediction accuracy of fourteen classical M_r_ prediction models was comparatively analyzed, from which the improved M_r_ prediction model incorporating gradation and stress variables was proposed for TRW-derived UPAB materials and further validated by external database accordingly. The results show that under the same *G/S* value and confining pressure level, the higher the SSR is, the greater the final M_r_ values are, and the more significant the effect of *G/S* on M_r_ is. Under the same SSR level, the increase of confining pressure alleviates the effect of *G/S* on M_r_. There appears to exist an optimal *G/S* value of around 1.6–1.8 that yields the best M_r_ behavior of the TRW-derived UPAB materials studied. The improved M_r_ prediction model was verified extensively to be universally applicable. It can potentially contribute to balancing long-term mechanical stability and drainability of TRW-derived UPAB materials through gradation optimization. The findings could provide a theoretical basis and technical reference for cost-effective and sustainable applications of UPAB materials derived from TRWs.

## 1. Introduction

In recent years, the frequency of occurrence and severity of damage of catastrophic weather events, including heavy precipitation and urban flooding (known as the El Niño phenomenon), have increased significantly around the global [1,2,3]. In order to mitigate flooding risks and related damages, the initiative of sponge cities has been widely enforced, where the construction of permeable pavements has emerged as one of the effective alternatives to enhance drainage capacity of urban road networks [4,5,6]. As compared to traditional impervious or low-permeability pavement structures constructed with dense-graded materials, permeable pavement layers constructed with open-graded materials feature larger porosity and better-connected pore structure, as well as the resulting advantages of improved drainability, skid resistance, and sound-absorbing and noise-reducing abilities [7,8,9,10,11]. As tunneling rock wastes (TRWs) are being increasingly produced and stockpiled, the sustainable recycling and reuse of such open- or gap-graded waste materials in permeable pavement construction becomes a major socioenvironmental concern and thus deserves in-depth research efforts. The majority of the existing research studies in the literature focused on the permeability, stability, and durability of permeable surface courses such as open-graded friction courses (OGFCs) [12], pervious Portland cement concrete (PCC) courses [13], porous asphalt concrete (AC) courses, and permeable wearing courses [14], while relatively few research studies were conducted on unbound permeable aggregate base (UPAB) materials with open gradation and high porosity for use underneath AC or PCC surface courses [15], particularly those for use in unsurfaced low-volume roads [16]. As a critical pavement layer, UPAB materials, in essence, serve the three major functionalities (denoted as “3Ps” for brevity), i.e., providing uniform and stable support for surface layers, protecting underlying subbase layers and/or subgrade soils, and promoting rapid drainage of excessive water out of pavement structures. However, high porosity and adequate permeability of UPAB often lead to insufficient bearing capacity and deformation stability [17], and previous studies barely resolved the challenging task of maximizing the drainage performance while maintaining desired mechanical stability. Resilient modulus (M_r_) of UPAB materials reflects the deviator stress-resilient strain relation, which is typically nonlinear and stress-dependent in nature, under the repeated applications of moving wheel loading [18,19]. It has become an important mechanical property and input parameter for mechanistic pavement design [20,21]. Therefore, it is of great significance to investigate resilient modulus characteristics of UPAB materials for further extending their use in higher-volume roads where their mechanical stability and durability are as important as drainage performance.

Generally speaking, there currently exists three main types of methods for determining M_r_ of unbound granular materials including UPAB materials, i.e., indirect back-calculation from falling weight (or lightweight) deflectometer measurements [22], direct measurement from laboratory repeated load triaxial tests [23], and indirect estimation from empirical or mechanistic–empirical predictive models [24]. As compared to the back-calculation method with heavy field-testing workload and uncontrollable field uncertainties and errors, the determination of M_r_ from laboratory repeated load triaxial tests is the most accurate and can capture the sensitivity of M_r_ to stress states and physical conditions (e.g., gradation, moisture, and compaction levels). The mechanistic–empirical M_r_ prediction models established accordingly from laboratory triaxial testing results are increasingly used as well.

Previous studies on laboratory repeated load triaxial tests of M_r_ of conventional dense-graded unbound granular materials were reported in the literature [25,26,27]. It was found that quantifying the influences of critical factors such as gradation, stress state, and moisture content on M_r_ is indispensable. The particle shape, aggregate source, and the maximum particle size (*D_max_*) were reported to significantly affect the mechanical properties of unbound aggregate base materials [28]. Greater M_r_ values were observed for unbound aggregate specimens containing coarser particles as compared to specimens containing finer particles [29]. The M_r_ values of angular aggregates with rough surfaces are greater than those of rounded aggregates with smooth surfaces [30]. The influence of stress states on M_r_ is complex and often mixed with those of material physical properties such as gradation, particle shape, moisture content, and degree of compaction [31]. Previous studies showed that M_r_ is influenced primarily by stress states, e.g., it increases significantly with increasing confining pressure [32,33,34,35,36]. It also increases with increasing loading frequency. When the loading frequency is low, M_r_ increases rapidly and then gradually transits to increase steadily with increasing frequency [37]. As the amplitude of cyclic deviator stress increases, M_r_ first increases linearly and then gradually transits to increase steadily [38]. As compared to stress states, material physical properties play a relatively secondary role in M_r_ of unbound granular materials. As seen from the above survey of the related literature, the strong dependency of M_r_ of conventional unbound granular materials on stress states and physical properties has been well studied in the past few decades; however, few studies have been conducted to characterize resilient modulus behavior of UPAB materials or optimize their gradations for balancing resilient modulus and drainage performance.

Despite that M_r_ characteristics of UPAB materials could be accurately obtained from laboratory repeated load triaxial tests, it is not practically affordable to run such time-consuming, labor-intensive tests for routine pavement design on a daily basis with widely varying combinations of major influential factors exhausted (e.g., gradation, stress state, and moisture content), partly because such tests require costly triaxial apparatus and well-trained personnel. Therefore, a variety of purely empirical correlations, complex elastoplastic constitutive models, and mechanistic–empirical predictive models have been developed in the literature to estimate M_r_ with different levels of accuracy. The initial M_r_ prediction models merely considered the influence of one single factor (e.g., confining pressure *σ_3_*, deviatoric stress *σ_d_*, or the first principal stress invariant I) [39,40,41], among which the one proposed by Seed [42] based on the first principal stress invariant was a particular example. Although Seed’s model is simple in its formula and applications, it still has the dimension problem. The M_r_ prediction models considering multiple factors were subsequently proposed, of which a few well-known examples can be named herein. Uzan [43] proposed an M_r_ prediction model considering both the first principal stress invariant and the deviatoric stress. It resolved the dimension problem, but encountered the new problem of indeterminate M_r_ value for zero octahedral shear stress. Ni et al. [44] further modified Uzan’s model [43] by adding the constant of 1 to both confining pressure and deviatoric stress terms, which not only improved the accuracy of the model but also solved the problems of indeterminate modulus value and the modulus dimension. Those single-factor or two-factor M_r_ prediction models were then applied in many studies [45,46,47], where the majority of them were found to be applicable only for fine-grained soils and only a few of them were applicable for unbound aggregate base materials [45,48,49]. Dan et al. [50] carried out a series of cyclic loading triaxial tests, quantitatively analyzed the water sensitivity of M_r_ of unbound aggregate materials under different stress states, and then established an improved nonlinear M_r_ prediction model. Ekblad [29] reported that the modified Uzan (*K-θ*) model is the best-fitting model for unbound granular materials. Among the existing M_r_ prediction models, few of them addressed gradation variations, particularly nontraditional open gradations. Therefore, it becomes indispensable to bridge such knowledge gaps.

In this study, five different TRW-derived UPAB gradations were first designed according to the gravel-to-sand ratio (*G/S*) concept proposed based on particle packing theory, along with the conventional dense gradation for comparison. Second, different combinations of confining pressure and cyclic deviator stress were generated by controlling the levels of the shear stress ratio (*SSR*), followed by laboratory repeated load triaxial tests under different stress paths. Note that the SSR is defined as the ratio of induced shear stress to shear strength of a specific granular material of interest, and its permanent deformation behavior can be allegedly controlled by limiting the SSR value [51,52]. Third, the influences of gradation variations and stress states on M_r_ characteristics of such TRW-derived UPAB materials were quantitatively analyzed. Finally, the prediction accuracy of fourteen different flagship M_r_ prediction models surveyed from the literature was compared and analyzed for all the 60 TRW-derived UPAB specimens tested. Accordingly, the modified M_r_ prediction model incorporating both gradation and stress state parameters was proposed, with its accuracy verified by external laboratory testing database. The findings could provide theoretical basis and technical reference for cost-effective and sustainable applications of TRW-derived UPAB materials in lower-carbon-emission, permeable pavement construction.

## 2. Materials and Laboratory Testing Program

### 2.1. Materials Tested

**Figure 1:**
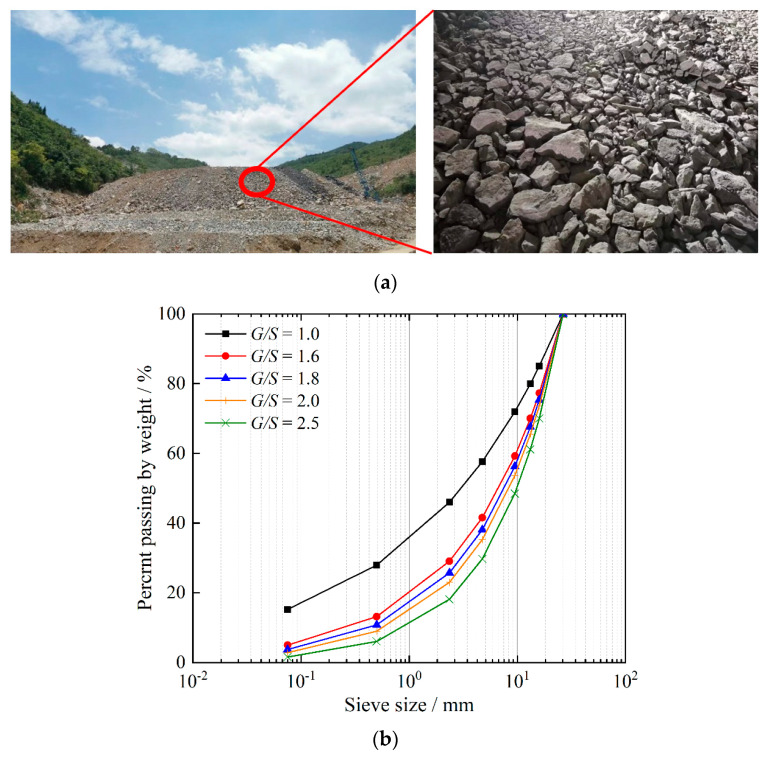
Illustration of (**a**) TRW-derived unbound aggregate materials and (**b**) gradation curves tested in the laboratory.

The unbound aggregate materials used in the laboratory tests, as shown in Figure 1a, were sampled from a disposal facility of tunneling rock wastes in the suburb of Changsha, Hunan Province, and the lithology of these materials is limestone. A total of five different gradations were designed and controlled using the gravel-to-sand ratio (*G/S*) concept proposed previously [53] to represent UPAB materials with varying particle-packing structures and thus permeability levels. It is worth noting that the *G/S* concept was proposed based on the packing theory and has been verified extensively for its applicability to unbound granular materials, of which more details can be found elsewhere [53]. The *G/S* parameter calculated accordingly via Equations (1) and (2) was found to control key mechanical properties (e.g., shear strength and permanent deformation) of unbound aggregates reasonably well [53]. Note that the parameters *D_max_* and n in Equation (1) are fitted from the gradation data by the Talbot function in Equation (2), where all the sieve sizes and related percentages of materials passing those sieves ought to be used.

(1) $$\frac{G}{S}=\frac{1-P_{4.75}}{1-\left(1-P_{4.75}\right)-P_{0.075}}=\frac{D_{\max}^{n}-4.75^{n}}{4.75^{n}-0.075^{n}}$$

(2) $$P_{i}={\left(\frac{d_{i}}{D_{\max}}\right)}^{n}$$

where $P_{i}$ is the percentage of materials passing the *i*-th sieve (%); $d_{i}$ is the opening aperture size of the *i*-th sieve (mm); $D_{max}$ is the maximum particle size (mm); and *n* is the shape parameter of the particular gradation curve, and it can be calculated from the given *G/S* value as per Equation (1), or be fitted from the gradation data by the Talbot function in Equation (2). It is worth noting that the *n* value was obtained by using the former method in this paper.

In order to follow related Chinese design codes [54] and avoid size effects for triaxial tests [55,56], the maximum particle size was determined to be 26.5 mm. In this study, a suitable gradation band with a maximum particle size of 26.5 mm was chosen, which is also qualified for use as unbound permeable aggregate gradations according to the Chinese technical specifications for highway pavement base construction [54,57]. Within this chosen gradation band, the corresponding *G/S* values range from 1.57–2.8. As plotted in Figure 1b, a total of five different gradation curves were selected from this gradation band to prepare TRW-derived UPAB specimens, and their *G/S* values were calculated to be 1.0, 1.6, 1.8, 2.0, and 2.5, respectively. The gradation curves with the *G/S* values of 1.6, 1.8, 2.0, and 2.5 represented low-, medium-, and high-level permeability, whereas the one with the *G/S* value of 1.0 served as the baseline against which the other four were compared due to its extremely low permeability. The *G/S* range of 1.0–2.5 studied is believed to well cover typical gradations of unbound aggregates used in pavement base construction practices. The related parameters of the gradation design scheme are summarized in Table 1.

### 2.2. Laboratory Compaction Tests

**Figure 2:**
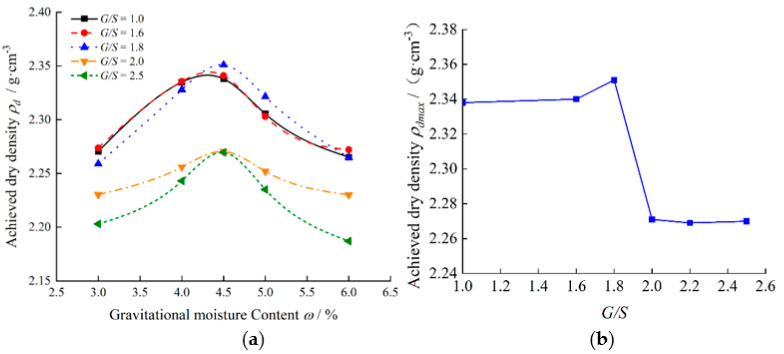
Laboratory compaction curves of unbound aggregate specimens: (**a**) achieved dry density versus moisture content and (**b**) achieved dry density versus *G/S*.

The TRW-derived unbound aggregate specimens with the *G/S* values of 1.0, 1.6, 1.8, 2.0, and 2.5 were prepared at five different initial moisture content levels of 3%, 4%, 4.5%, 5%, and 6% to carry out laboratory Proctor compaction tests, respectively. The inner diameter and height of the compaction mold were 150 mm and 120 mm, respectively. According to the Chinese geotechnical standards [57], the specimens were compacted in 3 sub-layers, with each sub-layer subjected to 98 blows. The applied compaction energy per unit volume was 2677 kJ/m^3^, which is similar to the 2693.3 kJ/m^3^ of the modified Proctor compaction according to AASHTO T180 [58]. The compaction curves of gravitational moisture content versus achieved dry density for specimens with different *G/S* values were obtained and are plotted in Figure 2, from which their optimal moisture content and maximum dry density values were determined accordingly.

It can be seen from Figure 2 that the compaction curves of the unbound aggregate specimens with different *G/S* values all exhibit similar trends, i.e., the achieved dry density first increases and then decreases with increasing moisture content, with clear peaks observed. The optimum moisture content value of the specimens with different *G/S* values is around 4.5%, according to Figure 2. However, the maximum dry density values of specimens with different *G/S* values are also different. As the *G/S* value gradually increases from 1.0 to 2.5, the maximum dry density of the specimens first increases and then decreases. There seems to exist a peak value of the maximum dry density of the specimens, of which the corresponding *G/S* value is between 1.6 and 1.8. This is consistent with the result reported by Zhou et al. [59]. This is expected, as fine fractions dominate for low *G/S* values and coarse fractions dominate for high *G/S* values. Therefore, specimens with either low or high *G/S* values are relatively more difficult to compact, thus justifying the existence of the peak value of maximum dry density.

### 2.3. Monotonic Triaxial Compression Tests

The triaxial test equipment is of the model No. STD and manufactured by the XIAN LETRY Company, Xi’an, China. The dimensions of the laboratory triaxial specimens were 100 mm in diameter and 200 mm in height with the diameter-to-height ratio of 1:2. The unbound aggregate specimens with different *G/S* values were fabricated at their respective optimal conditions (defined by the maximum dry density and related optimal moisture content) and then compacted in 5 sub-layers to achieve the degree of compaction of at least 98%. The degree of compaction is defined as the ratio of achieved dry density to the related maximum dry density at the same or similar compaction energy level. The compacted specimens were restrained with 0.5 mm thick latex membranes and then transferred to the triaxial chamber for subsequent laboratory monotonic and repeated load triaxial compression tests.

**Figure 3:**
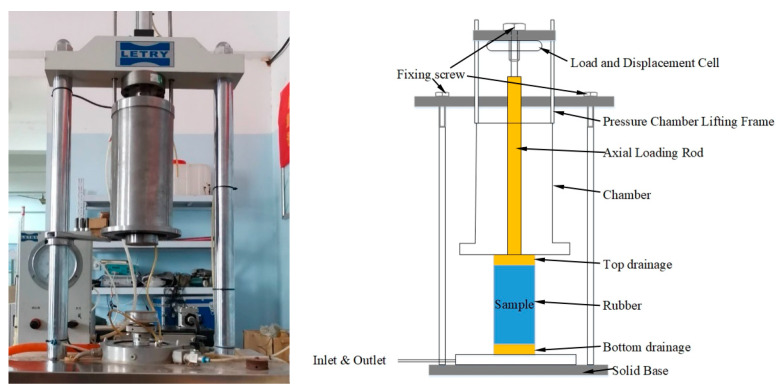
The apparatus used for laboratory monotonic and repeated load triaxial compression tests.

**Figure 4:**
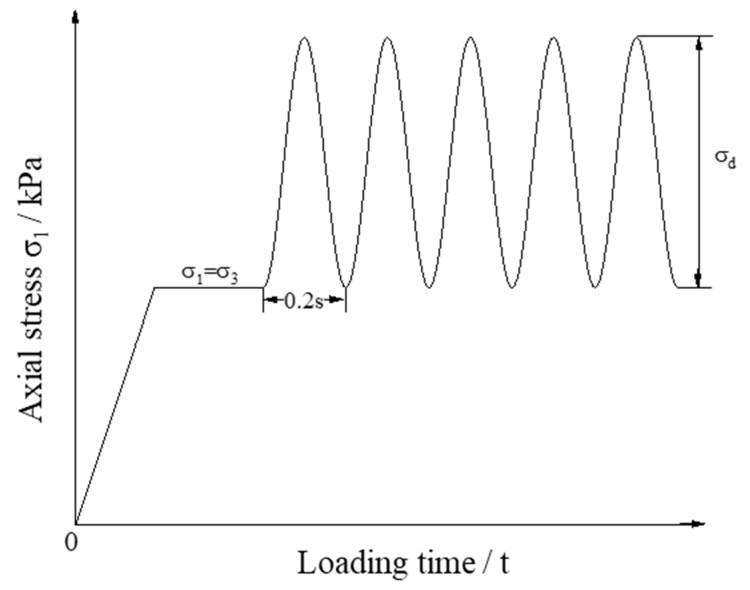
Illustration of the time–history curve of the axial load applied during laboratory repeated load triaxial compression tests.

The triaxial testing apparatus is shown in Figure 3. It is capable of applying both monotonic and repeated axial loads of different amplitude and/or frequency levels. The vertical deformation is recorded externally with the linear variable differential transducers (LVDTs). Each aggregate specimen was loaded under three different levels of confining pressure (σ_3_) of 50 kPa, 100 kPa, and 150 kPa, respectively. The half-sine waveform with a loading frequency of 5 Hz was adopted for deviator stress (*σ_d_*), as shown in Figure 4. The shear strength parameters (i.e., the apparent cohesion c and the angle of internal friction *ϕ*) of the specimens were determined by fitting the results of monotonic load triaxial compression tests against the classical Mohr–Coulomb failure criterion. 

### 2.4. Repeated Load Triaxial Tests

**Figure 5:**
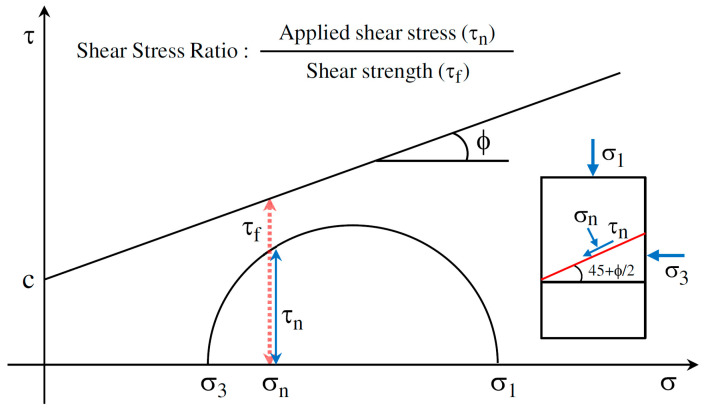
Illustration of the definition of the shear stress ratio (*SSR*).

**Figure 6:**
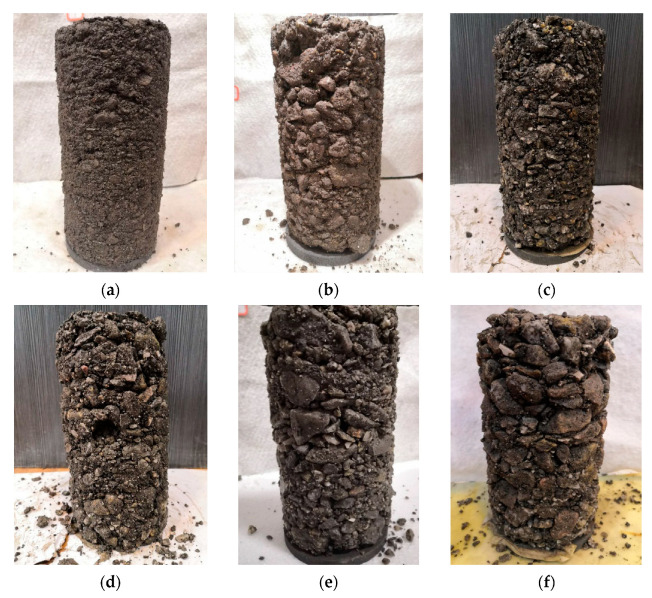
The real TRW-derived UPAB specimens with different *G/S* values upon the completion of 50,000 load applications during laboratory repeated load triaxial tests: (**a**) *G/S* = 1.0, (**b**) *G/S* = 1.6, (**c**) *G/S* = 1.8, (**d**) *G/S* = 2.0, (**e**) *G/S* = 2.2, and (**f**) *G/S* = 2.5.

**Figure 7:**
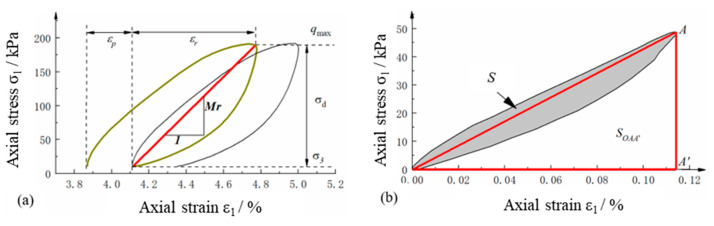
Illustration of the definitions of (**a**) resilient modulus and (**b**) damping ratio from axial stress–axial strain loops obtained from laboratory repeated load triaxial tests.

The repeated load triaxial (RLT) tests were further conducted to study the resilient and plastic strain behavior of TRW-derived UPAB materials, i.e., resilient modulus and permanent deformation characteristics. The shear stress ratio (*SSR*), of which the definition is illustrated in Figure 5, was used to control the amplitudes of cyclic deviator stress (*σ_d_*). The related calculation formulas are shown in Equations (3)–(6) sequentially [23]. To study the effect of stress level on resilient modulus of UPAB materials, four different SSR levels were designed for repeated load triaxial tests, i.e., 0.3, 0.5, 0.7, and 0.9, respectively. Therefore, each of the five different gradations corresponds to a total of 12 different combinations of stress states (i.e., 3 different σ_3_ levels times 4 different SSR levels), as listed in Table 2. The permanent deformation of the UPAB materials tended to be stable after 2000 load applications [53,55]. The dynamic properties of the UPAB materials were basically stable when the number of load applications exceeded 5000 [56]. By considering the stress levels applied to the specimens and the capacity of the triaxial testing apparatus, it was determined in this study that the maximum number of repeated load applications was set as 50,000. The specimens of five different gradations subjected to 50,000 load applications under 50 kPa confining pressure and 93. -kPa deviator stress were photographed and shown as examples in Figure 6.

(3) $$SSR=\frac{\tau_{f}}{\tau_{\max}}$$

(4) $$\tau_{f}=\frac{\sigma_{d}}{2}\cos\varphi$$

(5) $$\tau_{\max}=\sigma_{f}\tan\varphi +c$$

(6) $$\sigma_{f}=\sigma_{3}+\frac{\sigma_{d}\left(1-\sin\varphi \right)}{2}$$

(7) $$M_{r}=\frac{\sigma_{d}}{\varepsilon_{r}},\;\lambda =\frac{S_{\mathrm{cycle}}}{S_{{\mathrm{OAA}}^{\prime }}}$$

where $M_{r}$ is resilient modulus, $\varepsilon_{r}$ is resilient (or recoverable) axial strain, $\varepsilon_{p}$ is irreversible (or plastic) axial strain, λ is damping ratio, $S_{cycle}$ is the enclosed area of the axial stress–axial strain hysteresis loop, and $S_{OAA^{\prime }}$ is the enclosed area of the triangle $OAA^{\prime }$, as shown in Figure 7.

## 3. Results and Analysis

### 3.1. Plastic Strain Characteristics

**Figure 8:**
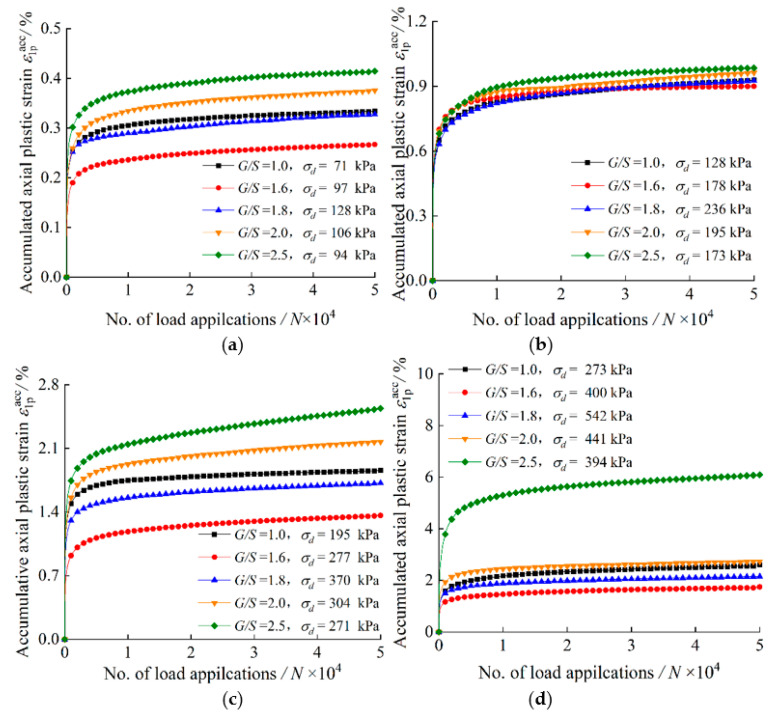
Accumulated axial plastic strain versus the number of load applications for TRW-derived UPAB specimens with varying *G/S* levels under 50 kPa confining pressure for (**a**) *SSR* = 0.3, (**b**) *SSR* = 0.5, (**c**) *SSR* = 0.7, and (**d**) *SSR* = 0.9.

Figure 8 shows the variation of accumulated axial plastic strain with the number of load applications (*N*) for TRW-derived UPAB specimens with different *G/S* levels under 50 kPa confining pressure. It is worth noting that the amplitudes of deviator stress (*σ_d_*) applied on the specimens with different *G/S* values are different even under the same combination of confining pressure and SSR due to their differences in shear strength. The accumulated axial plastic strain of the specimens increases rapidly with increasing number of load applications (*N*), while the rate of increase decreases during the initial loading stage. The accumulated axial plastic strain tends to be stable approximately at lower SSR levels (≤0.5), while it accumulates rapidly with increasing number of load applications (*N*) at higher SSR levels (≥0.7). The accumulated axial plastic strain increases significantly with increasing SSR under the same confining pressure and gradation. 

**Figure 9:**
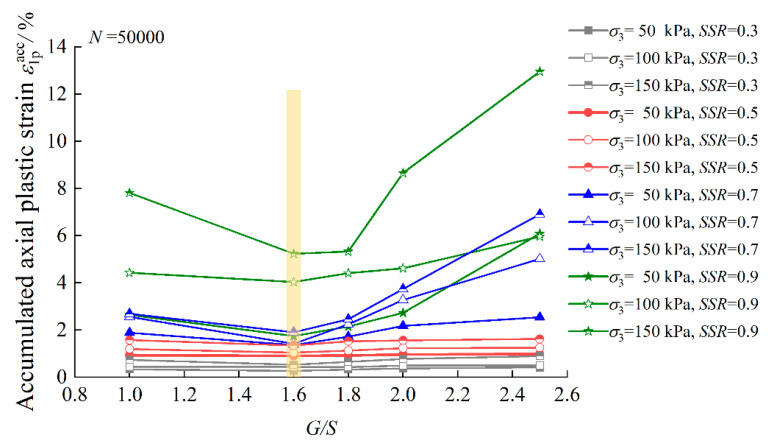
Accumulated axial plastic strain values upon *N* = 50,000 versus *G/S* levels for TRW-derived UPAB specimens under 50 kPa confining pressure.

In order to further study the influence of the gradation parameter *G/S* on the accumulated axial plastic strain (*ε_1p_^acc^*) of TRW-derived UPAB materials under different combinations of *σ_3_* and *SSR*, the ε_1p_^acc^ values calculated upon the completion of laboratory repeated load triaxial (RLT) tests (i.e., *N* = 50,000) are plotted for comparison in Figure 9. It can be seen from Figure 9 that under the same combination of *σ_3_* and *SSR*, the ultimate accumulated axial plastic strain first increases and then decreases with increasing *G/S* level. This implies that the ultimate accumulated axial plastic strain reaches its minimum and maximum values at *G/S* = 1.6 and *G/S* = 2.5, respectively. Therefore, the trend of the ultimate accumulated axial plastic strain varying with *G/S* is consistent with previously presented trends of maximum dry density and shear strength varying with *G/S*, respectively.

### 3.2. Resilient Modulus Characteristics

**Figure 10:**
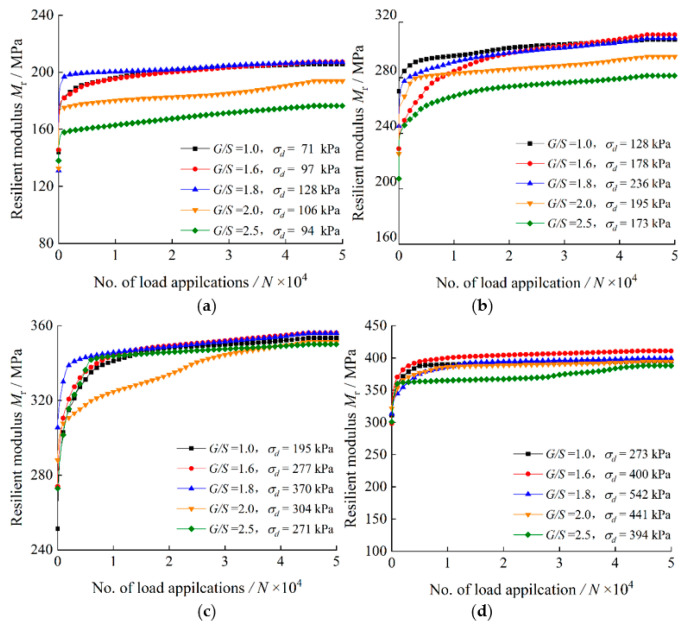
Resilient modulus versus the number of load applications for TRW-derived UPAB specimens with varying *G/S* levels under 50 kPa confining pressure for (**a**) *SSR* = 0.3, (**b**) *SSR* = 0.5, (**c**) *SSR* = 0.7, and (**d**) *SSR* = 0.9.

Figure 10 shows the variations of M_r_ with the number of load applications (*N*) for TRW-derived UPAB specimens with different *G/S* levels under 50 kPa confining pressure. It can be seen from Figure 10 that M_r_ increases rapidly with increasing number of load applications (*N*) at the initial loading stage until the gradually decreasing rate of increase becomes stable approximately at *N* = 1500. In addition, the M_r_ values of UPAB specimens all increase significantly with increasing SSR level, but still vary among different *G/S* levels even under the same stress state. The UPAB specimen with *G/S* = 2.5 exhibits the lowest M_r_ values, which may be attributable to its high fraction of coarse particles and large pores; in contrast, the UPAB specimen with *G/S* = 1.6 exhibits the greatest M_r_ values due to its relatively densest packing structure. Figure 10 also shows that the higher the SSR level is, the more significant the influence of gradation on M_r_ of UPAB specimens is, and the more pronounced the increases in M_r_ become. 

**Figure 11:**
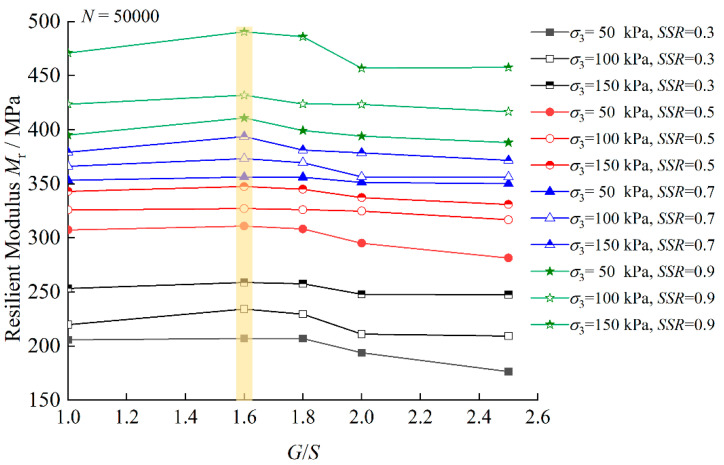
Resilient modulus values upon N = 50,000 versus *G/S* levels for TRW-derived UPAB specimens under 50 kPa confining pressure.

Figure 11 further illustrates the influence of the gradation parameter *G/S* on M_r_ characteristics of TRW-derived UPAB materials under different combinations of σ_3_ and SSR, where M_r_ values calculated upon N = 50,000 are plotted as examples. It can be seen from Figure 11 that under the same combination of σ_3_ and SSR, M_r_ values first increase and then decrease with increasing *G/S* level, thus clearly indicating that the highest M_r_ is reached at *G/S* = 1.6 and the lowest M_r_ at *G/S* = 2.5. Therefore, it appears that the optimal *G/S* value of 1.6–1.8 exists where the maximum resilient modulus (or the best resilient behavior) can be expected for UPAB materials. Under the same confining pressure level of 150 kPa and the varying SSR levels of 0.3, 0.5, 0.7, and 0.9, M_r_ values of the specimen with *G/S* = 1.6 increase by 4.53%, 4.99%, 5.95%, and 7.18% as compared to their counterparts of the specimens with *G/S* = 2.5, respectively. The influence of gradation on M_r_ characteristics of UPAB materials appears more pronounced for higher *SSR* levels. In addition, under the same SSR level of 0.5, as confining pressure increases, M_r_ values of the specimens with *G/S* = 1.6 increase by 7.64%, 5.24%, and 4.99% as compared to their counterparts of the specimens with *G/S* = 2.5, respectively. This implies that under the same *SSR* level, increasing confining pressure would alleviate the effect of gradation on resilient modulus of unbound permeable aggregate base materials, which is expected by the common engineering sense.

## 4. Development of Resilient Modulus Prediction Model

### 4.1. Comparative Assessment of Different Calculation Methods of Representative M_r_ Values

In the past few decades, resilient modulus prediction models for subgrade soils and unbound granular materials have evolved dramatically from primitive to sophisticated ones with key physical properties and the shearing and confinement effects considered sequentially. Among the existing M_r_ models in the literature, fourteen classical ones were shortlisted for comparative assessment in this study, as listed in Table 3. Since the matrix suction was not scoped into or measured during the laboratory testing program, those M_r_ prediction models incorporating matrix-suction-related terms were excluded from the scope of this study.

In order to study the influence of different calculation methods of the representative M_r_ values on the prediction accuracy of the M_r_ models, three different methods for calculating the representative M_r_ values were comparatively assessed. The first method averaged M_r_ values from the last 5 of the first 1500 load applications. The second method averaged M_r_ values from the last 10 of the first 10,000 load applications, whereas the third one averaged M_r_ values from the last 100 of the first 50,000 load applications. The entire sets of such representative M_r_ data calculated by using three different methods for UPAB specimens tested under different combinations of *G/S* levels and stress states were fitted against each of the M_r_ prediction models listed in Table 3. The resulting goodness-of-fit results (e.g., *R^2^* values) obtained for the fourteen different M_r_ prediction models according to the three different calculation methods of the representative Mr values are summarized in Table 3 as well.

Table 3 shows that the *R^2^* values of the fourteen different M_r_ models for the calculation method I are slightly greater than those for the calculation methods II and III. Overall speaking, little difference is observed among such three different methods in terms of the prediction accuracy, thus indicating that any one of them is suitable for calculating representative M_r_ values in this study. By comparing the *R^2^* values of the fourteen different M_r_ models, it further proves that the addition of both standard atmospheric pressure (Pa) and the constant of 1 has little effect on the improvement of prediction accuracy, and that the inclusion of additional model coefficients k_4_ and k_5_ is not effective for improving the prediction accuracy of the fourteen different M_r_ prediction models.

### 4.2. Comparative Assessment of Existing Classical M_r_ Prediction Models

The M_r_ prediction models listed in Table 3 were employed to fit the laboratory-measured representative M_r_ results under different combinations of *G/S* levels and stress states. The goodness-of-fit indicators including the coefficient of determination (*R^2^*), adjusted *R^2^* (adj. *R*^2^), and root mean square error (RMSE) were calculated for comparative assessment of such different M_r_ models. Since the axial strain (or deformation) of the specimens recorded during each load application of laboratory RLT tests consists of both resilient and plastic parts, the resilient strain induced by each load application can be used to calculate M_r_ and evaluate its variation with the number of load applications under different combinations of *G/S* and stress levels, whereas the plastic strain can be used to evaluate permanent deformation accumulation behavior. To facilitate such comparisons, the above-mentioned method I was adopted herein to calculate the representative M_r_ values of each specimen. The results of such representative M_r_ values were then fitted by each of the M_r_ prediction models listed in Table 3 to assess their applicability and accuracy of prediction. The specimens with *G/S* = 1.0 were taken as examples to illustrate this process and the related results in the following subsections. Although not presented herein for brevity, the comparison results of laboratory-measured versus model-predicted representative M_r_ values for UPAB specimens with other *G/S* values than 1.0 (i.e., *G/S* = 1.6, 1.8, 2.0, or 2.5) also exhibit similar trends and observations to be detailed subsequently.

**Figure 12:**
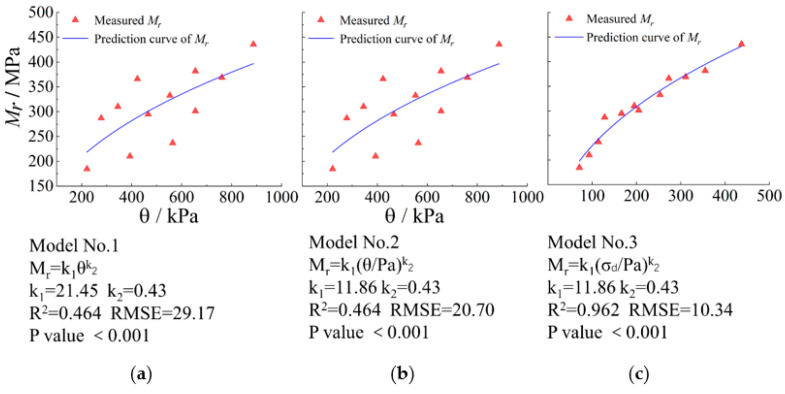
Comparisons of laboratory-measured representative M_r_ values against predicted values by different univariate M_r_ prediction models for TRW-derived UPAB specimens with *G/S* = 1.0: (**a**) Model No. 1, (**b**) Model No. 2, and (**c**) Model No. 3.

Figure 12 plots the 12 sets of laboratory-measured representative M_r_ data for the specimens with *G/S* = 1.0, along with the results predicted by classical univariate M_r_ models. It can be seen from Figure 12 that the *R^2^* value is only 0.464 for both Model No. 1 and Model No. 2 incorporating the first principal stress invariant (*θ*) only. This indicates that the prediction accuracy of both Models No. 1 and No. 2 is poor, and that normalizing the stress variable by the standard atmospheric pressure barely improves model accuracy. In contrast, the *R^2^* value of Model No. 3 incorporating deviatoric stress ($\sigma_{d}$) only reaches 0.962, thus indicating that the prediction accuracy is relatively satisfactory. It further proves that the correlation between *M*_R_ and $\sigma_{d}$ is more significant than θ for UPAB materials studied.

**Figure 13:**
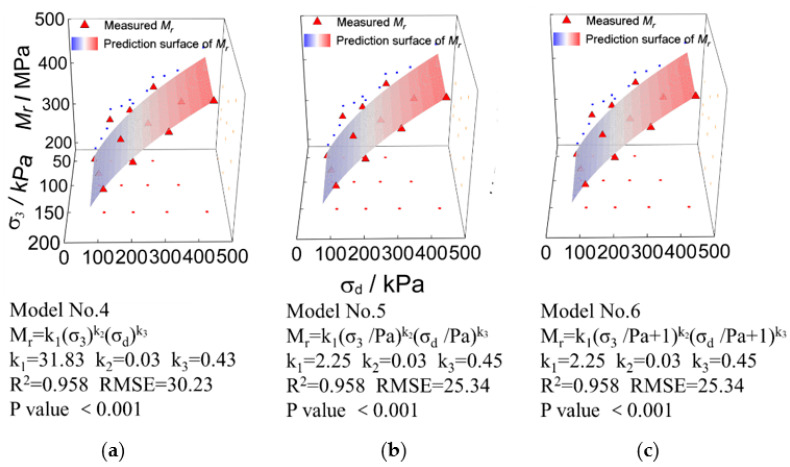
Comparisons of laboratory-measured representative M_r_ values against predicted values by different bivariate (i.e., $\sigma_{3}$ and $\sigma_{d}$) models for TRW-derived UPAB specimens with *G/S* = 1.0: (**a**) Model No. 4, (**b**) Model No. 5, and (**c**) Model No. 6.

The comparisons of laboratory-measured representative M_r_ values against predicted values by different bivariate (i.e., $\sigma_{3}$ and $\sigma_{d}$) M_r_ prediction models are shown in Figure 13 for UPAB specimens with *G/S* = 1.0. It can be observed from Figure 13 that the *R^2^* values of those bivariate M_r_ prediction models (i.e., Model No. 4, Model No. 5, and Model No. 6) incorporating both $\sigma_{3}$ and $\sigma_{d}$ are all above 0.95 and insignificantly different from each other. Due to the dimension inconsistency of Model No. 4, it was abandoned and excluded from further analysis. By comparing the *R^2^* values between M_r_ prediction models No. 5 and No. 6, it can be found that the addition of the constant of 1 to stress variables in Model No. 6 results in almost negligible difference in prediction accuracy.

**Figure 14:**
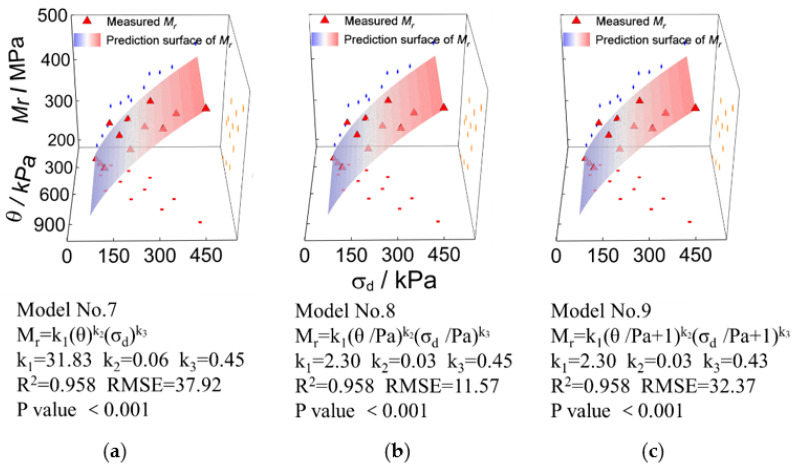
Comparisons of laboratory-measured representative Mr values against predicted values by different bivariate (i.e., *θ* and $\sigma_{d}$) models for TRW-derived UPAB specimens with *G/S* = 1.0: (**a**) Model No. 7, (**b**) Model No. 8, and (**c**) Model No. 9.

Figure 14 shows the comparisons of laboratory-measured and model-predicted representative M_r_ values for the UPAB specimens with *G/S* = 1.0. The M_r_ prediction models compared are bivariate (i.e., *θ* and $\sigma_{d}$) as well. It can be seen from Figure 14 that the *R^2^* values of those bivariate M_r_ prediction models (i.e., Model No. 7, Model No. 8, and Model No. 9) are all above 0.95. Similar to the bivariate models incorporating $\sigma_{3}$ and $\sigma_{d}$ (i.e., Model No. 6), the addition of the constant of 1 to the stress variables *θ* and $\sigma_{d}$ in Model No. 9 also results in little improvement in the accuracy of model prediction.

**Figure 15:**
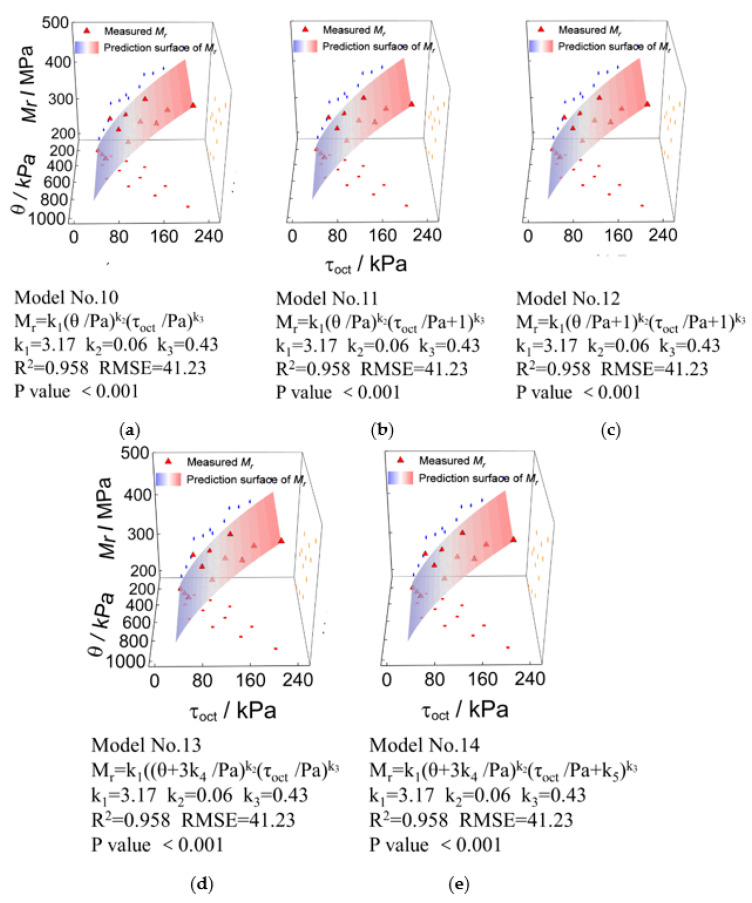
Comparisons of laboratory-measured representative M_r_ values against predicted values by different bivariate (i.e., *θ* and $\tau_{oct}$) models for TRW-derived UPAB specimens with *G/S* = 1.0: (**a**) Model No. 10, (**b**) Model No. 11, (**c**) Model No. 12, (**d**) Model No. 13, and (**e**) Model No. 14.

Similarly, the laboratory-measured representative M_r_ values and those predicted by other prediction models incorporating the first principal stress invariant (*θ*) and the octahedral shear stress ($\tau_{oct}$) (i.e., Models No. 10 to No. 14) are compared and plotted in Figure 15 for the UPAB specimens with *G/S* = 1.0. It can be seen from Figure 15 that the *R^2^* values of Models No. 10, No. 11, and No. 12 are consistent, thus indicating that the addition of standard atmospheric pressure (Pa) and the constant of 1 has little effect on improving the prediction accuracy. However, the *R^2^* values of Models No. 14 and No. 15 slightly decrease as compared to those of Models No. 10 to No. 12, indicating that the addition of model coefficients *k*_4_ and *k*_5_ is not effective for accuracy improvement. 

### 4.3. Selection of the Optimal Classical M_r_ Prediction Model

**Figure 16:**
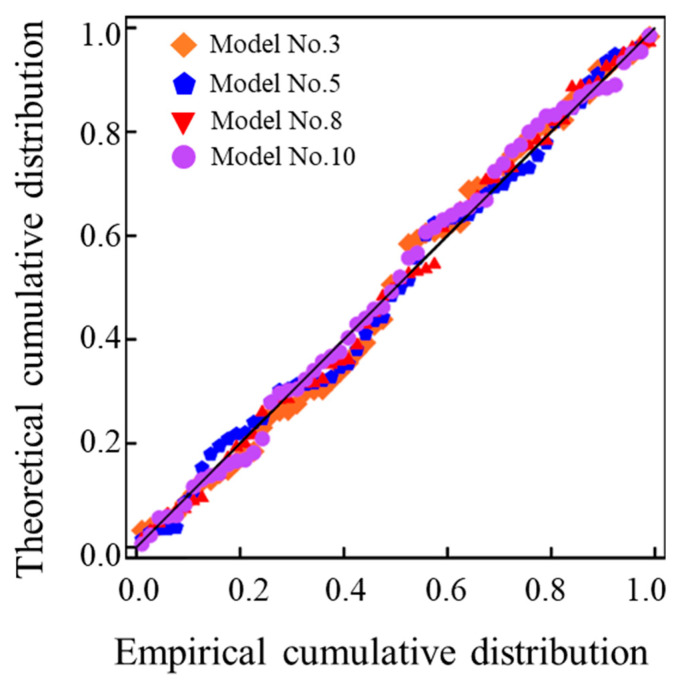
The P–P diagrams of standardized residuals of the four M_r_ prediction models evaluated.

The aforementioned M_r_ prediction models with *R^2^* values greater than 0.95 were further shortlisted to perform additional assessment. Due to the dimension inconsistency existing in the formulas of Models No. 4 and No. 7, they were excluded from further analysis. As presented previously, the addition of standard atmospheric pressure (Pa) and the constant of 1 has little effect on the improvement of prediction accuracy, and the inclusion of additional model coefficients k_4_ and k_5_ reduces the prediction accuracy. Therefore, the focus was to further compare Models No. 3, No. 5, No. 8, and No. 10 through comprehensive statistical analysis. Table 4 summarizes the statistics of the residual eigenvalues between the laboratory-measured representative M_r_ values and their counterparts predicted by Models No. 3, No. 5, No. 8, and No. 10 for the entire UPAB specimens, respectively. Model No. 8 is observed to have the lowest residual sum of squares, variance, root mean square error (RMSE), and Akaike information criterion (AIC) index, as compared to the other three models. In addition, the skewness of Model No. 8 is the lowest and its kurtosis is relatively large, thus indicating that its residual distribution is the closest to the normal distribution and its goodness-of-fit is the best. Therefore, in terms of the overall prediction accuracy, Model No. 8 is the best among the four M_r_ models evaluated for the UPAB materials studied. Figure 16 plots the P–P diagrams of the normalized residuals between the measured and predicted representative M_r_ values. Note that the P–P diagrams were drawn according to the relations between the cumulative proportions of predicted M_r_ values and those of the normal distributions. That is, when the dataset conforms to the normal distribution, each data point approximately falls on the straight line of normal distribution in the P–P graph. It can be found from Figure 16 that the normal P–P diagrams of the standardized residuals generated by the four M_r_ prediction models all coincide approximately with the straight line. The P–P diagram of Model No. 8 is much closer to the straight line than those of other models. This further confirms that the goodness-of-fit of Model No. 8 is the best among those four models evaluated.

Hence, the optimal prediction model was determined to be Model No. 8. It was then used to fit the measured representative M_r_ data of the UPAB specimens tested under different combinations of *G/S* levels and stress states in the laboratory repeated load triaxial tests. The model coefficients k_1_, k_2,_ and k_3_ and related adjusted coefficient of determination (Adj. *R^2^*) were determined for each *G/S* level accordingly and are listed in Table 5. It can be seen from Table 5 that as the *G/S* value increases, the Adj. *R^2^* value decreases but is still greater than 0.85. The values of the model coefficient k_1_ are quite different among different *G/S* values, while the values of model coefficients k_2_ and k_3_ show insignificant difference with more concentrated distributions.

### 4.4. Modification of the Optimal M_r_ Prediction Model

As shown previously, the values of the three model coefficients k_1_, k_2,_ and k_3_ of the optimal M_r_ prediction Model No. 8 are considerably different from each other. In order to further address the influences of gradation variation (quantified by varying *G/S* levels), statistical correlation analysis was performed between the gradation control parameter *G/S* and the fitted model coefficients k_1_, k_2_, and k_3_. Different types of fitting functions (e.g., linear, quadratic, power, and logarithmic) were attempted, respectively. It was found by trial and error that there exists a statistically significant quadratic function relating model coefficient k_1_ and the gradation parameter *G/S*, and a power function relating k_2_ and *G/S*. However, k_3_ and *G/S* are not statistically correlated. Therefore, Equations (8) and (9) were obtained accordingly, thus leading to the modified version of the M_r_ prediction Model No. 8, as formulated in Equation (10). Note that the model coefficient k_3_ in the M_r_ prediction Model No. 8 varied insignificantly among different *G/S* levels and thus was eventually set as the constant of 0.405, the average k_3_ value from different *G/S* levels.

k_1_ = 0.421(G/S)^2^ − 1.6895(G/S) + 3.552 (8)

(9) $$k_{2}=0.0629{\left(G/S\right)}^{1.0432}\;$$

(10) $$M_{r}=\left(0.421{\left(G/S\right)}^{2}-1.6895\left(G/S\right)+3.552\right)P_{a}{\left(\frac{\theta }{P_{a}}\right)}^{0.0629{\left(G/S\right)}^{1.0432}}{\left(\frac{\sigma_{d}}{P_{a}}\right)}^{0.405}\;$$

where *M_r_* is resilient modulus, k_1_ and k_2_ are model coefficients to be regressed, *G/S* is the gradation control parameter, $\sigma_{d}$ is deviatoric stress (kPa), θ is the first principal stress invariant (kPa), and Pa is the standard atmospheric pressure.

**Figure 17:**
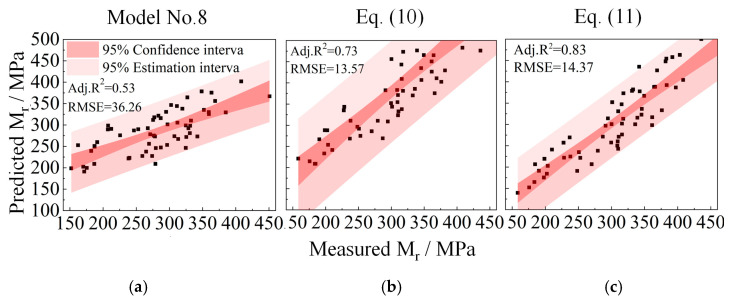
Comparison of the laboratory-measured representative M_r_ values against those predicted by (**a**) the initial Model No. 8 without considering gradation variations, (**b**) Equation (10), and (**c**) Equation (11).

In order to substantiate the validity and accuracy of the above-modified optimal M_r_ prediction model (see Equation (10)), its predicted representative M_r_ values were compared against the laboratory-measured counterparts of a total of 60 UPAB specimens, as shown in Figure 17b. It can be clearly seen from Figure 17b that the prediction errors increase with increasing M_r_ values, and that the prediction errors for greater M_r_ values are mainly attributable to the specimens with *G/S* values of 2 and 2.5. Therefore, it becomes imperative to further improve Equation (10) by dividing it by the arithmetric square root of the gradation parameter *G/S*, as shown in Equation (12).

(11) $$M_{r}=\left[0.421{\left(G/S\right)}^{1.5}-1.6895{\left(G/S\right)}^{0.5}+3.552{\left(G/S\right)}^{-0.5}\right]P_{a}{\left(\frac{\theta }{P_{a}}\right)}^{\left[0.0629{\left(G/S\right)}^{1.0432}\right]}{\left(\frac{\sigma_{d}}{P_{a}}\right)}^{0.405}$$

The representative M_r_ values of all the UPAB specimens predicted by Equation (11) were compared against their laboratory-measured counterparts accordingly. Figure 17c shows that the resulting *R^2^* value of Equation (11) increases from 0.73 to 0.83 as compared to Equation (10), indicating that the modification made to Equation (10) (i.e., Equation (11)) is effective in terms of enhancing the M_r_ prediction accuracy for UPAB materials. The prediction accuracy of the original M_r_ Model No. 8 is shown in Figure 17a as well, where the R^2^ value only reaches 0.53 and is much lower than that yielded by the modified model (i.e., Equation (11)). Therefore, the modified model formulated in Equation (11) was finalized as the optimal M_r_ prediction model.

### 4.5. Parametric Sensitivity Analysis of the Final Optimal M_r_ Predictive Model

**Figure 18:**
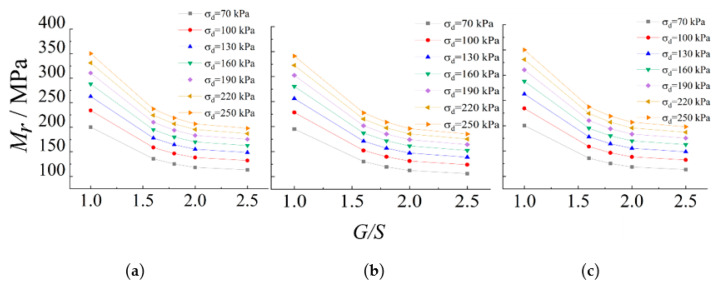
The sensitivity of representative M_r_ to gradation variation (quantified by the parameter G/S) evaluated from Equation (11) under (**a**) 50-kPa, (**b**) 100-kPa, and (**c**) 150-kPa confining pressure levels, respectively.

The sensitivity of M_r_ to gradation variations under different stress states was studied by using the final optimal Mr prediction model (i.e., Equation (11)). The results of such parametric sensitivity analysis are plotted in Figure 18. It can be seen from Figure 18 that for *G/S* values within the range of 1.0–2.5, the representative M_r_ value predicted by Equation (11) decreases with increasing *G/S* value under the same combination of confining pressure and deviatoric stress, that is, as the content of fine particles decreases, the representative M_r_ values decrease. In addition, the gradation variation has a much greater influence on the representative M_r_ for lower *G/S* values. This implies that the representative *M_r_* is more sensitive to the gradation variation for UPAB materials with higher fraction of fine particles. In contrast, the gradation variation has little effect on the representative M_r_ for relatively large *G/S* value, i.e., the representative *M_r_* is less sensitive to the gradation variation for the UPAB materials with higher fraction of coarse particles.

## 5. Verification of the Developed Resilient Modulus Prediction Model

The additional external resilient modulus database was collected from the literature [67,68] to further verify the previously developed and validated M_r_ prediction model (i.e., Equation (11)). The three different types of commonly-used unbound aggregate materials were tested, i.e., uncrushed gravel, crushed limestone, and crushed dolomite. Four different levels of fines content (i.e., materials passing No. 200 or 0.075 mm sieve) were designed for each type of unbound aggregate materials, i.e., 4%, 8%, 12%, and 18%. They correspond to the *G/S* values of 1.39, 1.43, 1.49, and 1.55, respectively. In addition, two different types of fines, i.e., plastic (clay with plasticity index of 10–20%) and nonplastic (mineral powder with plasticity index of near zero), were used for each type of unbound aggregate materials as well. Although each type of unbound aggregate materials was tested under three different levels of moisture content (i.e., 90% × w_opt_, w_opt_, and 110% × w_opt_) to study the influence of moisture content, only the M_r_ data measured at the optimal conditions were selected for use in the model verification, as the final optimal M_r_ prediction model was previously developed using the M_r_ data measured at the optimal conditions only in this study. Table 6 summarizes the basic physical properties of the three different types of unbound aggregate materials tested in the literature [67,68].

By using the above-mentioned external M_r_ datasets, Table 7 compares the laboratory-measured M_r_ values against those predicted by the final optimal model established in Equation (11) for each of the three different types of unbound aggregate materials. It can be seen from Table 7 that the M_r_ prediction model established in this study for UPAB materials is also reasonably accurate for the external unbound aggregates with different combinations of mineralogy, fines content, and plasticity index of fines. Overall, it works better for either limestone aggregate materials or aggregate materials with plastic fines as compared to other materials. Note that the model coefficients in Equation (11) need to be calibrated (or updated) due to the differences in specific physical properties between the external materials from the literature and those tested in this study. Therefore, to further improve the prediction accuracy, the model coefficients related to the *G/S* parameter were set to be regressed, thus yielding Equation (12).

(12) $$M_{r}=\left[m_{1}{\left(\frac{G}{S}\right)}^{1.5}+m_{2}{\left(G/S\right)}^{0.5}+m_{3}{\left(G/S\right)}^{-0.5}\right]P_{a}{\left(\frac{\theta }{P_{a}}\right)}^{\left[m_{4}{\left(G/S\right)}^{m_{5}}\right]}{\left(\frac{\sigma_{d}}{P_{a}}\right)}^{m_{6}}$$

where *M_r_* is resilient modulus; $m_{1}$, $m_{2}$, $m_{3}$, $m_{4}$, $m_{5}$, and $m_{6}$ are model coefficients to be regressed; *G/S* is the gradation control parameter; $\sigma_{d}$ is deviatoric stress; θ is the first principal stress invariant; and Pa is the standard atmospheric pressure.

**Figure 19:**
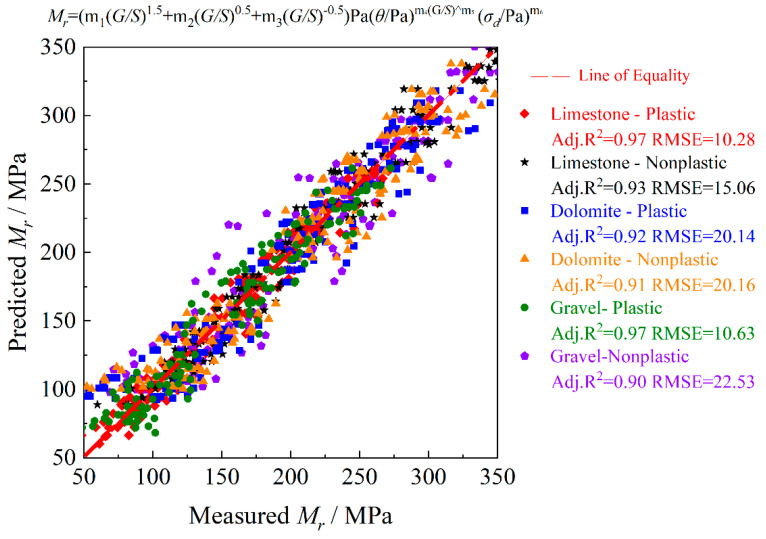
Comparison of measured and predicted M_r_ values of three different types of unbound aggregates in the external database for model verification.

The comparison results of laboratory-measured M_r_ values and those predicted by Equation (12) for the above-mentioned external M_r_ datasets are plotted in Figure 19. It shows that the prediction accuracy for the three different types of unbound aggregate materials is improved considerably, with Adj. *R^2^* values all greater than 0.90. Additionally, the prediction accuracy for limestone aggregate materials or those with plastic fines is relatively the best, which is consistent with the aforementioned results obtained by Equation (11). 

## 6. Summary and Conclusions

In this study, five different gradations were designed to study the gradation effect on resilient modulus behavior of TRW-derived UPAB materials. Laboratory repeated load triaxial tests were conducted under different combinations of confining pressure and cyclic deviator stress as controlled by the levels of the shear stress ratio (*SSR*). The M_r_ prediction model incorporating both gradation and stress state parameters was developed from all the 60 TRW-derived UPAB specimens tested, and the validity and applicability of the developed model were further verified by external laboratory testing database. The major findings of this study are summarized as follows.

(1) The M_r_ values of the TRW-derived UPAB materials studied increase rapidly with increasing number of load applications. Under the same *G/S* value and confining pressure level, the higher the shear stress ratio (*SSR*) is, the greater the final M_r_ values are, and the more significant the effect of gradation (*G/S*) on M_r_ is. Under the same SSR level, the increase of confining pressure alleviates the effect of gradation (*G/S*) on M_r_ of the TRW-derived UPAB materials studied. There appears to exist an optimal *G/S* value of around 1.6–1.8 that yields the best resilient modulus behavior of the TRW-derived UPAB materials studied.
(2) The addition of standard atmospheric pressure (Pa) and the constant of 1 in the M_r_ models has little effect on the improvement of prediction accuracy, whereas the inclusion of two additional model coefficients, k_4_ and k_5_, is not effective for improving prediction accuracy of the M_r_ models. Among the M_r_ prediction models compared, the bivariate Model No. 8 incorporating the first principal stress invariant (*θ*) and deviatoric stress (σ*_d_*) possesses the best prediction accuracy.
(3) By comparing and analyzing the existing M_r_ prediction models, a benchmark prediction model suitable for the TRW-derived UPAB materials studied was selected. The statistical correlations among the fitted model coefficients, the gradation control parameter *G/S*, and stress state variables were further analyzed, from which the improved M_r_ prediction model was proposed and verified extensively to be universally applicable.

## Figures and Tables

**Table 1:** Gradation design scheme and corresponding gradation parameters.

G/S	*D*_max_/mm	*n*	*C_u_*	*C_c_*
1.0	26.5	0.32	270.22	3.55
1.6	26.5	0.51	29.09	1.97
1.8	26.5	0.56	23.64	2.08
2.0	26.5	0.61	18.09	1.90
2.5	26.5	0.71	11.63	1.63

Note: *C_c_* is the coefficient of curvature; *C_u_* is the coefficient of uniformity.

**Table 2:** The loading scheme adopted for laboratory repeated load triaxial tests.

*G/S*	*c*/kPa	*φ*/°	Confining Pressure/kPa	*SSR*	Deviator Stress/kPa	No. of Load Applications (*N_max_*)
1.0	65.5	29.2	50	0.3/0.5/0.7/0.9	71/128/195/273	50,000
			100		93/166/253/355	
			150		114/205/311/273	
1.6	64.8	40.9	50		97/178/277/400	
			100		136/150/388/560	
			150		175/321/498/400	
1.8	64.3	47.9	50		128/236/370/542	
			100		184/341/535/793	
			150		241/446/699/542	
2.0	64	43.6	50		106/195/304/441	
			100		151/277/432/628	
			150		196/360/561/441	
2.5	48.7	45.5	50		64/173/271/394	
			100		142/261/407/592	
			150		189/348/543/394	

Note: The deviator stress values were calculated from the prescribed confining pressure and SSR values by using the obtained shear strength parameters (*c* and *ϕ*).

**Table 3:** The goodness-of-fit results of the fourteen different M_r_ prediction models based on the three different methods for calculating representative M_r_ values of each of the TRW-derived UPAB specimens.

M_r_ Prediction Model			Fitted Coefficient of Determination (*R*^2^)		
No.	Source	Formula	Method I	Method II	Method III
1	*K-θ* Model [42,60,61]	$M_{r}=k_{1}\theta^{k_{2}}$	0.46	0.43	0.42
2	Modification K-θ Model [42,60,61]	$M_{r}=k_{1}{\left(\frac{\theta }{P_{a}}\right)}^{k_{2}}$	0.46	0.43	0.42
3	Moossazadeh & Witczak Model [41]	$M_{r}=k_{1}{\left(\frac{\sigma_{d}}{P_{a}}\right)}^{k_{2}}$	0.96	0.95	0.95
4	Pezo Model [62]	$M_{r}=k_{1}{\left(\sigma_{3}\right)}^{k_{2}}{\left(\sigma_{d}\right)}^{k_{3}}$	0.96	0.96	0.95
5	Raad & Figueroa Model [63]	$M_{r}=k_{1}P_{a}{\left(\frac{\sigma_{3}}{P_{a}}\right)}^{k_{2}}{\left(\frac{\sigma_{d}}{P_{a}}\right)}^{k_{3}}$	0.96	0.96	0.95
6	Ni Model [44]	$M_{r}=k_{1}{\left(\frac{\sigma_{3}}{P_{a}}+1\right)}^{k_{2}}{\left(\frac{\sigma_{d}}{P_{a}}+1\right)}^{k_{3}}$	0.96	0.96	0.95
7	Uzan Model [43]	$M_{r}=k_{1}{\left(\theta \right)}^{k_{2}}{\left(\sigma_{d}\right)}^{k_{3}}$	0.96	0.96	0.96
8	Octahedral Shear Stress Model [43]	$M_{r}=k_{1}P_{a}{\left(\frac{\theta }{P_{a}}\right)}^{k_{2}}{\left(\frac{\sigma_{d}}{P_{a}}\right)}^{k_{3}}$	0.96	0.96	0.96
9	Modified Ni Model [44]	$M_{r}=k_{1}{P_{a}(\frac{\theta }{P_{a}}+1)}^{k_{2}}{\left(\frac{\sigma_{d}}{P_{a}}+1\right)}^{k_{3}}$	0.96	0.96	0.96
10	Modified Uzan Model [43]	$M_{r}=k_{1}P_{a}{\left(\frac{\theta }{P_{a}}\right)}^{k_{2}}{\left(\frac{\tau_{oct}}{P_{a}}\right)}^{k_{3}}$	0.96	0.96	0.96
11	NCHRP 1-28A Model [64,65]	$M_{r}=k_{1}P_{a}{\left(\frac{\theta }{P_{a}}\right)}^{k_{2}}{\left(\frac{\tau_{oct}}{P_{a}}+1\right)}^{k_{3}}$	0.96	0.96	0.96
12	Improved Ni Model [44]	$M_{r}=k_{1}{P_{a}(\frac{\theta }{P_{a}}+1)}^{k_{2}}{\left(\frac{\tau_{oct}}{P_{a}}+1\right)}^{k_{3}}$	0.96	0.96	0.96
13	Superpave Performance Model [66]	$M_{r}=k_{1}P_{a}{\left(\frac{\theta -3k_{4}}{P_{a}}\right)}^{k_{2}}{\left(\frac{\tau_{oct}}{P_{a}}\right)}^{k_{3}}$	0.95	0.94	0.94
14	Hopkins Model [66]	$M_{r}=k_{1}P_{a}{\left(\frac{\theta -3k_{4}}{P_{a}}\right)}^{k_{2}}{\left(\frac{\tau_{oct}}{P_{a}}+k_{5}\right)}^{k_{3}}$	0.95	0.94	0.94

Notes: $M_{r}$ denotes resilient modulus (MPa); $k_{1}$, $k_{2}$, $k_{3}$, $k_{4}$, and $k_{5}$ denote model coefficients to be fitted; θ denotes the first principal stress invariant (kPa), i.e., $\theta =\sigma_{1}+\sigma_{2}+\sigma_{3}$; $\tau_{oct}$ denotes the octahedral shear stress, i.e., $\tau_{oct}=\frac{1}{3}\sqrt{{\left(\sigma_{1}-\sigma_{2}\right)}^{2}+{\left(\sigma_{1}-\sigma_{3}\right)}^{2}+{\left(\sigma_{1}-\sigma_{3}\right)}^{2}}$ (or $\tau_{oct}=\frac{\sqrt{2}}{3}\sigma_{d}$ in triaxial stress space where $\sigma_{2}=\sigma_{3}$); and $P_{a}$ denotes the reference stress that is generally taken as the standard atmospheric pressure of 100 kPa.

**Table 4:** Comparison of the residual statistics of four M_r_ prediction models evaluated.

Statistics	Model No. 3	Model No. 5	Model No. 8	Model No. 10
Residual sum of squares	23,116.7	25,465.61	22,296.19	25,329.81
Variance	391.776	430.270	371.282	420.497
Root mean square error	19.79	20.74	19.26	20.51
Skewness	0.058	−0.024	−0.024	−0.187
Standard error of skewness	0.309	0.309	0.309	0.309
Kurtosis	−0.815	−0.367	−0.631	−0.383
Standard error of kurtosis	0.608	0.608	0.608	0.608
AIC index	361.23	369.04	361.07	368.72

**Table 5:** Regressed model coefficients and adjusted *R^2^* values of the optimal M_r_ prediction model No. 8 for TRW-derived UPAB specimens with varying *G/S* levels.

G/S Value	k_1_	k_2_	k_3_	Adj. *R*^2^
1.0	2.29569	0.06051	0.42957	0.95759
1.6	1.89589	0.0897	0.46408	0.95544
1.8	1.82463	0.15902	0.38588	0.85698
2.0	1.947	0.12619	0.36525	0.88606
2.5	1.93914	0.14658	0.38141	0.86677

**Table 6:** The basic physical properties of unbound base/subbase aggregate materials tested in the literature and used as additional external datasets for M_r_ model validation.

Unbound Aggregate Materials		Optimal Moisture Content/%	Maximum Dry Density/G·cm^3^	*G/S*	Plasticity Index (PI)/%
**Limestone with nonplastic fines**	L4NP	10.5	2.122	1.39	0
	L8NP	8.4	2.182	1.43	0
	L12NP	7.6	2.243	1.49	0
	L16NP	6.8	2.269	1.55	0
**Limestone** **with plastic fines**	L4P	9.3	2.165	1.39	10
	L8P	8	2.171	1.43	8
	L12P	7.6	2.229	1.49	9
	L16P	7.1	2.270	1.55	9
**Dolomite with nonplastic fines**	D4NP	9.2	2.114	1.39	0
	D8NP	8.1	2.147	1.43	0
	D12NP	7.1	2.198	1.49	0
	D16NP	7.1	2.208	1.55	0
**Dolomite with plastic fines**	D4P	8.0	2.149	1.39	11
	D8P	7.3	2.168	1.43	12
	D12P	7.1	2.194	1.49	13
	D16P	6.9	2.184	1.55	14
**Gravel with nonplastic fines**	G4NP	10.6	2.118	1.39	0
	G8NP	8.5	2.211	1.43	0
	L12NP	6.8	2.259	1.49	0
	G16NP	6.6	2.286	1.55	0
**Gravel with plastic fines**	G4P	10.4	2.157	1.39	8
	G8P	7.4	2.208	1.43	9
	G12P	6.6	2.242	1.49	9
	G16P	6.6	2.256	1.55	9

Notes: L denotes limestone; D denotes dolomite; G denotes gravel; P denotes plastic (clay); NP denotes nonplastic (mineral power); and the number in the symbol of each material denotes the fines content (i.e., percentage of materials passing No. 200 or 0.075 mm sieve), for example, “L4NP” denotes unbound crushed limestone aggregate materials with the nonplastic fines content of 4%.

**Table 7:** The prediction accuracy of the developed M_r_ model formulated by Equation (11) for the three different types of unbound aggregates in the external database.

Material Type	*R^2^*
**Limestone with plastic fines**	0.6575
**Limestone with nonplastic fines**	0.6098
**Dolomite with plastic fines**	0.6218
**Dolomite with nonplastic fines**	0.6161
**Gravel with plastic fines**	0.5874
**Gravel with nonplastic fines**	0.5841

## Data Availability

Some or all data, models, or code that support the findings of this study are available from the corresponding author upon reasonable request.
